# Hygiene in the Practice of Medicine

**Published:** 1859-12

**Authors:** V. H. Taliaferro

**Affiliations:** Atlanta, Ga.


### ARTICLE III.

*Hygiene in the Practice of Medicine.* By V. H. Taliaferro, M. D., Atlanta, Ga.

Hygiene is a subject which, in our opinion, should at this day command more attention than any one subject connected with the Medical Profession, and yet, perhaps, it is less regarded than any other. It does not confine itself to *eating* and *drinking*, to mental and physical culture, &c., but embraces a wide field, taking in all those great natural laws which govern and influence the universe. The revolutions of the earth with its varied seasons, bringing the genial air of Spring and heat of Summer, and gradually and imperceptibly, as it were, gliding into fall, until the chill winds of winter are sweeping the earth, purifying and invigorating the face of nature, night and day, the unspotted sky, with its undisturbed electric currents, as well as the clouds with their electrical phenomena, all have their controlling influences upon the human system. And these influences are of such character as must control in no little degree the progress and terminations of abnormal or pathological conditions. We lay down as an unfailing maxim that no natural law or principle can be violated without its evil consequence; the maintainance of health and the successful management of disease, therefore must depend, to no inconsiderable extent, upon their careful observance.

We will, in the first place consider in as condensed manner as possible, the influences of *Solar Light* upon health and disease.

Says a writer upon this subject "Man in his most perfect type is doubtless to be found in the temperate regions of the globe, where the social influences of light, heat and chemical rays are so richly balanced. Under the scorching rays of the tropics, man cannot call into exercise his highest powers. The caloric rays are all-powerful there, and lassitude of body and immaturity of mind aae the necessary results, while in the darkness of the polar regions the distinctive characters of our species almost disappear, in the absence of those solar influences which are so poweful in the orgrnic world. We perceive thus the influence of the different degrees of the sun's power over animal developement, and consequently infer that continued total darkness is incompatible with organic existence.

Solar light is stimulant and tonic. This we conclude from facts already mentioned, and from the fact that the germ cells of the invertebrata, as well as some of the lower order of vertebrata animals, are by the influence of the sun's rays developed into embryonic life; also the disappearance of corpuscles in the blood of individuals confined to dark localities. The stimulant action of light is perhaps more observant in the vegetable than animal kingdom, the fact of which is strikingly exemplified by the unfolding of flowers to the morning sun and closing again upon the approach of night. Plants reared in the dark are whitish, denoting a want of the coloring principles of their circulating fluids. So marked, indeed, is the stimulant properties of light upon vegetable growths that when confined to the dark they will bend towards a crevice through which is admitted a ray of light, as if to drink and relish its life-giving influence. We are told by Dr. Priestley that plants in the dark are in a state similar to sleep, and that they resume their functions when placed under the influence of light and the direct action of the solar rays. We mention these facts merely to show the analogy between the effect of solar light upon the animal and vegetable kingdoms. The most remarkable fact is, that in each, the circulating fluids are impoverished in its absence. What then does this argue? That darkness predisposes to the developement of disease; that debility and impoverished blood needs *especially* the influence of the sun's rays and that anæmia, enervated and impoverished constitutions, cannot be renovated in the dark. No practice is more prevalent among invalids than the exclusion from their chambers of the sun's light, and no practice is perhaps, more pernicious. Take for an example, an emaciated dyspeptic, whose blood is poor and thin; whose tissues have all wasted from deficient nutrition and in whom the functions are all lazily and languidly performed, and confine him for weeks to an ordinary invalid's chamber, in which the windows are closed and curtained with studied care, and the effect must necessarily be, notwithstanding the most skilful and judicious medication, a continued and persistent aggravation of all his difficulties. The consumptive will be upon his bed for months without for once feeling the life-giving and soul-enlivening influence of the clear sun light, while his physician is perchance filling his system with drugs for the enrichment of his blood and the building up of his tissues, when nature's own tonic and invigorator is excluded from him. Our confidence in the application of medicinal agents should not lead us into forgetfulness of these controlling natural agencies. Let the anæmic, the impoverished constitution, the dyspeptic and consumptive throw aside their dark window curtains and let freely flow the light of heaven in their dark chambers, and gloom and despondency will possess them less frequently, the blood will receive a new impetus, and flow more freely through the capillary system—its richness will be increased and the tissues and functions therefore invigorated. If these conclusions be true, and we believe our reasoning proves them so, are we, as medical practitioners sufficiently observant in giving our patients the advantages of solar light? As a general thing the Doctor leaves it with his patient whether his room be dark or light.

There are, of course, conditions in which the influence of the sun's light would be pernicious, as in acute inflammation, even active antiphlogistic measures are necessary to be adopted. In such cases darkness would be preferable, because it is the opposite of light and its influence is sedative. In most cases of sickness of whatever character the patient needs either the stimulant action of solar light or the sedative influence of darkness. One striking advantage to be derived from giving a patient an ordinary amount of sunlight, is that the system will be more readily cognizant of the sedative influence of the darkness of night, and quiet slumber therefore more certainly insured.

It has been observed in some of the large European hospitals that patients recovered less rapidly in the darker wards, and where the light was almost entirely excluded, the fatality was greatest. This is a fact of the greatest importance to the Medical Practitioner.

To sum up then our conclusions, we infer 1st. That light is essential to organic developement, and perfect conformation. 2d. That it is stimulant and tonic. 3d. That it enriches the blood and enlivens the vital actions. 4th. That darkness is the reverse of light and its influence is sedative.

Pure air consists of a relative proportion of oxygen, nitrogen and carbonic acid. It is impure whenever these proportions are destroyed, or when nnduly combined with other properties, and consequently deleterious to health. By an adult 19152 solid inches of atmospheric air is respired every hour, and 319 solid inches every minute. We therefore perceive the necessity of a strict attention to a perfect and continual ventilation of invalid apartments, for when the system is debilitated by disease, it of course loses more or less of its powers of resistance to morbific influences. The sick chamber is usually permitted to be crowded by friends and acquaintances; the atmosphere of which soon becomes, from its deficiency of oxygen, and impregnation with carbonic acid, unfit for use, and especially for an invalid. Yet how rarely does the physician, in his over-weaning anxiety to deal out his potions with a skillful hand, examine with scrutinizing care the department of his patient to know if the air he breathes is easily and readily exchanged for that which is pure. We have no question but physicians not unfrequently, in making their morning calls, are astonished to find that patients whom they expected strengthened and improved from their studied and skillful prescriptions, weaker and in a more precarious condition, when the fact is, the air of the room had, during the night, been breathed and re-breathed by the patient and his attendants until, loaded with carbonic acid, the already diseased and weakened system yields to the influence of poisoned air.

A diseased system needs especially a pure and uncontaminated atmosphere, and in consideration of this fact we deem it highly deleterious to have a number of attendants confined for days and nights within the sick chamber.

The sick patient not unfrequently has a bed-fellow, and almost invariably is the sick infant taken to bed at night upon the arm of its mother or its nurse, and breathes continually the impure and poisonous exhalations from her lungs. When such gross errors are permitted, medication can be of but little avail. Frequently *little* things turn the scale for either *life* or *death*, and we believe we are warranted in saying that frequently the balance is given in favor of the latter by the powerfully poisonous influences of carbonic acid exhaled from the Lungs of the sick and their attendants in closely confined sick chambers.

If we would eradicate disease and build up debilitated and enervated constitutions, let us give our patients, both night and day, a pure, cool atmosphere, with its due proportions of oxygen and nitrogen, giving life and vigor to the vital energies, and a perfect and natural transformation of venous to arterial blood. Every physician who understands the relationship between atmospheric air and organic existence, knows the truth of all we say, and yet how common to neglect them.

